# Predictive Artificial Intelligence Models for Mycotoxin Surveillance and Mitigation in Poultry Production Systems

**DOI:** 10.3390/toxins18090392

**Published:** 2026-09-10

**Authors:** Padmini Vaka, Laharika Kappari, Gokul V. Selvaraj, Ramesh K. Selvaraj, Todd J. Applegate, Revathi Shanmugasundaram

**Affiliations:** 1 Department of Poultry Science, University of Georgia, Athens, GA 30605, USA; 2 Toxicology and Mycotoxin Research Unit, U.S. National Poultry Research Center, Agricultural Research Service, U.S. Department of Agriculture, Athens, GA 30605, USA

**Keywords:** mycotoxins, artificial intelligence, machine learning, hyperspectral imaging, predictive modeling, control strategies, poultry feed safety

## Abstract

Mycotoxins are widespread contaminants in animal feeds and continue to pose serious threats to animal welfare, production efficiency, food security, and sustainable economic growth worldwide. Chickens are highly susceptible to multiple mycotoxins, such as aflatoxins (AFLs), deoxynivalenol (DON), fumonisins (FBs), zearalenone (ZEA), ochratoxin A (OTA), and T-2 toxin. Exposure to these toxins can damage intestinal integrity, compromise immune functions, impair nutrient absorption, and ultimately reduce production efficiency even at subclinical concentrations. Conventional mycotoxin detection methods, such as enzyme-linked immunosorbent assays (ELISA) and chromatographic techniques, provide accurate quantification but remain expensive, labor-intensive, time-consuming, and inefficient for large-scale screening, particularly when masked mycotoxins are present. The frequent co-occurrence of multiple mycotoxins further complicates risk assessment and effective management. Emerging analytical technologies, including hyperspectral imaging, biosensors, Internet of Things (IoT)-based platforms, and machine-learning algorithms, offer promising advancements for rapid detection and predictive risk forecasting. This review summarizes the toxicological impacts of major mycotoxins on poultry, outlines critical challenges in detection and prevention, and evaluates current artificial intelligence (AI) and machine learning (ML) approaches for mycotoxin identification, prediction, and management. A systematic literature search conducted across PubMed, ScienceDirect and Google Scholar identified 176 unique studies published between 2010 and 2026. Key knowledge gaps include limited availability of high-quality datasets and source code, inconsistent model interpretability, and poor reproducibility across production environments. By integrating conventional toxicology with data-driven approaches, this review highlights how predictive modeling can strengthen mycotoxin surveillance and support proactive mitigation strategies in modern poultry production systems.

## 1. Introduction

Mycotoxins are toxic secondary fungal metabolites ubiquitously present in agricultural crops and their by-products, posing substantial challenges across the globe [1]. Numerous climatic and environmental factors, including high temperature and humidity, excessive rainfall, inadequate ventilation, improper storage conditions, and poor hygiene, promote fungal growth and mycotoxin production [2]. Exposure to mycotoxins, whether acute or chronic subclinical concentrations, can lead to toxicity, carcinogenicity, decreased production performance, and significant welfare issues in livestock [3,4,5]. Beyond their biological effects, mycotoxins impose a significant economic burden through reduced crop quality and yield, increased disease prevention and biosecurity costs, food waste, and trade restrictions [6]. In the United States alone, annual crop losses associated with mycotoxin contamination are estimated at approximately USD 900 million, with an additional USD 400 million associated with testing and quality control processes [1,7]. Overall, these challenges emphasize the critical need for continuous surveillance and effective mitigation strategies.

Given these global challenges, non-ruminants, particularly swine and poultry, represent a major component of global meat and egg production and are highly susceptible to mycotoxin exposure [8]. Rapid expansion of the poultry sector has increased pressure on feed supply chains, resulting in more complex raw material sourcing and greater susceptibility to feed-related contamination [9,10]. Within this context, mycotoxins remain a persistent challenge because of their stability, ubiquity in grains, and difficulty to control under commercial production conditions [11]. As cereal grains and their by-products are common poultry feed ingredients, mycotoxin contamination is nearly unavoidable and poses a significant One Health concern affecting animal health and human food safety, and environmental safety across the farm-to-fork continuum [12,13].

Fungal genera such as *Aspergillus*, *Fusarium*, and *Penicillium* produce the major mycotoxins of concern that threaten poultry production. These risks are further exacerbated by the globalization of feed supply chains; dependence on imported feed ingredients during periods of domestic scarcity can introduce region-specific fungal strains that intensify existing contamination patterns and complicate quality control and preventive strategies [14,15]. Adding further complexity, feed ingredients such as maize and wheat, as well as processed feeds, are frequently contaminated with multiple toxins simultaneously [7,15,16]. Even at low concentrations, these combinations of multiple mycotoxins can exert additive or synergistic effects, causing more severe physiological damage to birds than individual toxins such as DON and FBs alone [17,18]. Chronic mycotoxin exposure carries significant economic consequences because overt clinical signs, including beak erosions or fatty liver, often do not manifest during subclinical exposure, allowing birds to appear healthy yet experience impaired digestion, compromised immune function and overall decreased production performance [19]. Since feed costs comprise 60–70% of total production cost, even small losses in growth or feed efficiency from subclinical mycotoxicosis can tighten already narrow profit margins and negatively affect farm profitability.

Beyond these effects on poultry, mycotoxins pose direct risks to human health due to residue accumulation in meat, eggs, and visceral organs. Tolerable daily intake limits are extremely low, including 0.25 µg/kg body weight (BW) per day for ZEA, 1 µg/kg BW/day for DON and its modified forms, and a provisional limit of 2 µg/kg BW/day for FBs [20]. Even trace levels transferred into poultry products may exceed these thresholds, emphasizing the need for modern preventive strategies aimed at minimizing residual contamination. Therefore, advanced preventive approaches, including predictive modeling, have become increasingly essential for protecting both animal and human health within modern poultry production systems [21].

Despite the severity of the issue, current mycotoxin management strategies rely heavily on conventional detection methods, and interventions typically occur only after contamination has already occurred. Immunoassays and chromatographic techniques remain the most commonly used diagnostic tools [22], while high-performance liquid chromatography (HPLC) and liquid chromatography combined with tandem mass spectrometry (LC-MS/MS) represent the gold standard for quantifying mycotoxins [23,24,25]. However, these methods require specialized equipment, trained personnel and high analytical costs, which limits their practicality for rapid, large-scale screening in globalized feed supply chains [12].

To address these limitations, the increasing adoption of digital tools in agriculture has led to the emergence of digital toxicology, an interdisciplinary field that integrates toxicology with AI and ML. These technologies enable early forecasting of contamination risks, enhance surveillance, and support timely interventions before mycotoxins enter livestock feed or the human food chain [26]. In poultry systems, AI and ML provide opportunities to transition from traditional detection-only approaches toward integrated approaches that leverage both conventional methods and ML-based predictive tools for proactive risk assessment and improved protection. This review provides the first poultry-focused synthesis of established and emerging AI/ML applications for mycotoxin detection, prediction, and control. It integrates information from conventional toxicology, spectral and sensor-based technologies, and data-driven modeling to outline how predictive tools can strengthen surveillance within modern poultry production systems.

Current applications of machine-learning tools for mycotoxin monitoring remain limited by several constraints. Existing models are rarely validated for use in commercial poultry-feed systems, and most have been developed using single-grain datasets or focus on individual parent toxins. Limited attention has been given to compound feeds, naturally occurring co-contamination, masked mycotoxins, or relationships between feed contamination and poultry-level physiological responses. Many available datasets are small or geographically restricted, lack prospective or external validation, and provide limited information on model sensitivity, robustness, and practical applicability. It is also important to note that the application of machine-learning approaches for mycotoxin surveillance and prevention in poultry remains an emerging area with limited validated models, restricted datasets, and few examples of integration into commercial decision-making, which represents a key limitation in the current literature. In response to these gaps, this review aims to (i) summarize major mycotoxins affecting poultry and their toxicological impacts; (ii) outline conventional detection and mitigation strategies currently used in poultry production; (iii) critically evaluate established and emerging AI and ML approaches for mycotoxin identification, prediction, and monitoring; and (iv) identify practical limitations and future priorities for developing reliable, interpretable, and field-ready predictive tools to support decision-making in feed mills, farms, and regulatory surveillance programs.

## 2. Review Methodology

This review examined peer-reviewed studies published from 2010 to 2026. A structured literature search was conducted in PubMed, ScienceDirect and Google Scholar over a six-month period, which provided 650 unique studies related to mycotoxins in feed and food. Principal search terms such as names of major mycotoxins (AFL or DON or FBs or ZEA or OTA or T-2 toxin or HT-2 toxin or masked mycotoxin) combined with poultry-specific and machine-learning terms (poultry, feed, mitigation, deep learning, neural network, random forest, support vector machine, and gradient boosting). Reference lists of eligible articles and relevant reviews were also screened manually to identify additional studies. Inclusion criteria were peer-reviewed English-language articles reporting mycotoxin effects in poultry, detection methods, or conventional and machine learning model-based mitigation strategies. Exclusion criteria included non-peer-reviewed sources, and articles lacking clear information on analytical limits, detection ranges, data type, or model validation. After screening full articles, 176 studies met the eligibility criteria and were retained for detailed evaluation.

## 3. Overview of Major Mycotoxins of Concern in Poultry Health

**Figure 1:**
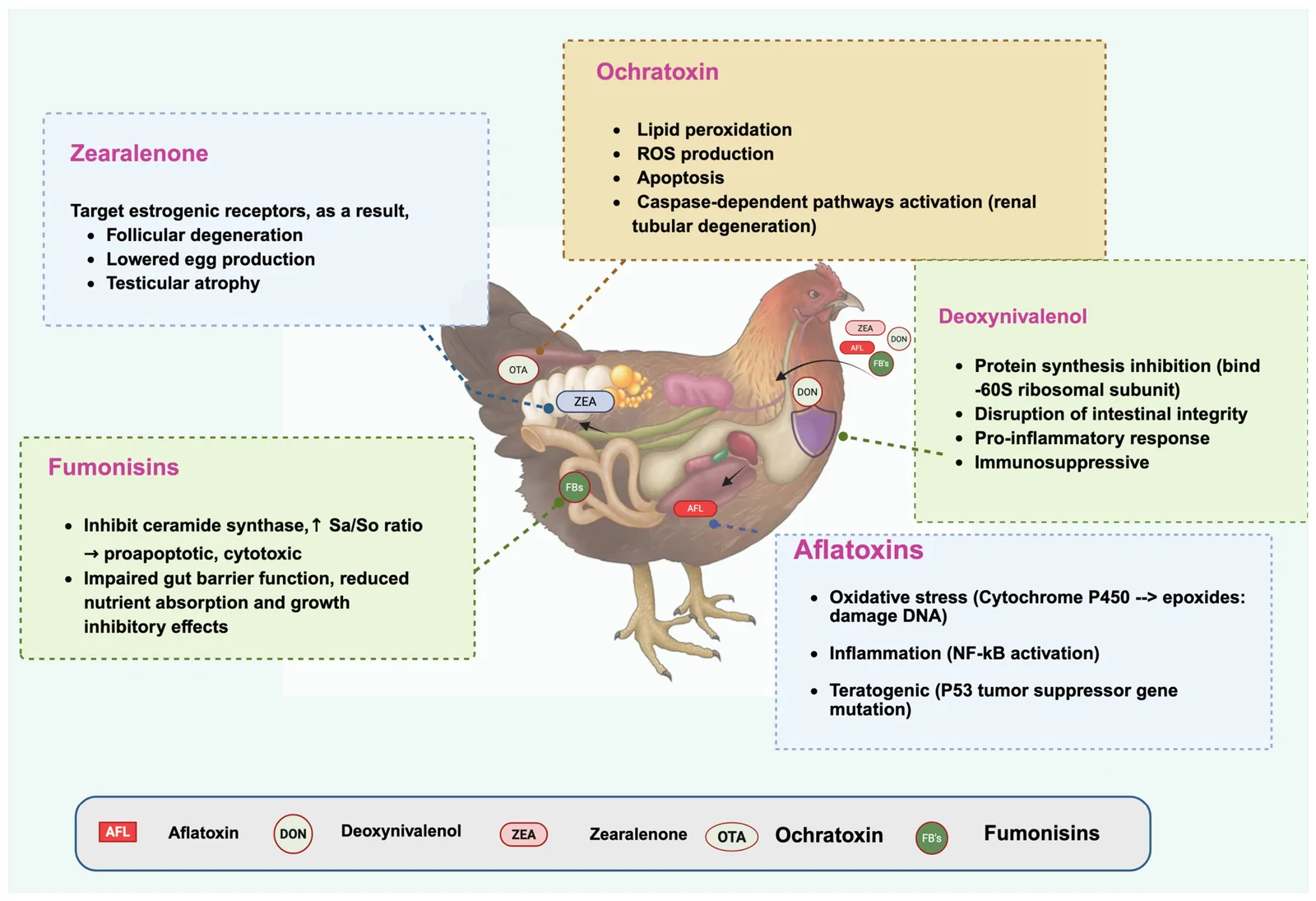
Impact of major mycotoxins on cellular and molecular pathways. Created in BioRender. Publication License: Shanmugasundaram, R. (2026) https://BioRender.com/zhojocn accessed on 15 June 2026.

Poultry exposure to mycotoxins results from a complex interaction between birds’ susceptibility and the ongoing trends in feed contamination [19]. Because commercial poultry diets rely heavily on cereal grains and agro-industrial by-products, birds are frequently exposed to diverse fungal metabolites that can impair physiological systems even at low concentrations [23]. These mycotoxins exert their toxic effects through several interconnected biological mechanisms, as illustrated schematically in Figure 1.

### 3.1. Aflatoxins

AFLs, primarily aflatoxin B_1_ (AFB_1_), are produced by *Aspergillus flavus* and *Aspergillus parasiticus,* and are known for their potent hepatotoxic mycotoxins affecting poultry [13,27]. Regulatory limits for AFLs in poultry feed are strict, reflecting the high sensitivity of birds to these toxins. According to United States Food and Drug Administration (FDA) and European Union (EU) guidelines, the maximum permissible concentrations of AFL in poultry feed are 0.1 mg/kg for mature birds and 0.02 mg/kg for birds younger than eight weeks, which aligns with the general EU limit of 0.02 mg/kg [19]. These thresholds reflect the high sensitivity of poultry AFL exposure and the severity of associated clinical outcomes.

AFL exposure contributes to marked reductions (10–25%) in body weight gain (BWG) and a 15–30% increase in feed conversion ratio (FCR), along with increased susceptibility to infectious diseases and carcinogenic effects [5,17,28] in chickens. Following ingestion, AFB_1_ interacts with cytochrome P450 enzymes and is bio-transformed into the reactive metabolite AFB_1_-8,9-epoxide [29]. This metabolite readily binds to DNA and forms adducts, initiating a cascade of biochemical changes that impair detoxification pathways, alter cellular metabolism and increase the risk of mutagenesis and tumor development [30]. At the cellular level, AFB_1_ interferes with protein synthesis, induces oxidative stress, and triggers cytotoxicity, DNA damage, and apoptosis [15]. Necropsy findings commonly reveal hepatomegaly and pale liver indicative of fatty degeneration [31]. Correspondingly, histopathological examination shows focal hemorrhages, sinusoidal congestion, cytoplasmic vacuolation, and, in severe cases, necrosis, bile duct proliferation, lymphoid infiltration and fibrosis [5].

At the molecular level, broilers exposed to 1 mg/kg of AFB_1_ exhibit decreased expression of antioxidant enzymes such as superoxide dismutase (SOD) and increased expression of inflammatory mediators, including toll-like receptor 4 (TLR4), Nuclear factor kappa light chain enhancer of activated B cells (NF-κB), tumor necrosis factor alpha (TNF-α), inducible nitric oxide synthase (iNOS), Cytochrome P450 Family 1 Subfamily A Member 1 (CYP1A1), Interleukin 1 Beta (IL-1β), Interleukin 6 (IL-6), and Interleukin 8 (IL-8) [17,32,33]. AFL contamination also poses significant public health concerns. As ingested AFB_1_ and residues can be transferred to poultry tissues, approximately 1/1200 of the dietary concentration transfers to meat and 1/2200 transfers to eggs [28,34]. Although these values are low, they may still contribute to human exposure in regions with weak regulatory oversight or inadequate grain quality control.

Environmental conditions strongly influence AFL occurrence. Aspergillus species thrive in warm, humid environments, making AFB_1_ contamination more prevalent in warmer seasons and tropical regions [34,35]. As global feed supply chains increasingly depend on international grain markets, strict regulatory monitoring of imported commodities is essential, particularly those sourced from regions where hot and humid conditions complicate storage and handling [15].

### 3.2. Deoxynivalenol

DON, produced primarily by *Fusarium graminearum*, is one of the most prevalent trichothecene mycotoxins in poultry feed and remains a major concern due to its stability during milling and thermal processing [36]. As a type B trichothecene, DON binds to the 60S ribosomal subunit and inhibits protein synthesis. This interaction activates mitogen-activated protein kinase (MAPK) signaling pathways, leading to leukocyte apoptosis and suppression of innate immune responses. Higher toxin concentrations trigger macrophage apoptosis and further weaken host defenses [37]. Regulatory agencies have established strict limits because of the strong sensitivity of chickens to DON. The FDA and European Union recommended a maximum concentration of 5 mg/kg in broiler chicken diets [7,38], reflecting its potential to impair growth and disrupt gastrointestinal health even at moderate doses.

The small intestine is the primary target of DON toxicity; DON exposure reduces nutrient absorption and causes a 10–18% reduction in BWG. DON damages the intestinal lining, reduces villus length and width, and alters the expression of tight junction proteins such as claudins [28,34]. Disruption of gut barrier integrity increases intestinal permeability, promotes dysbiosis, and compromises immune defense, contributing to enteric diseases including subclinical necrotic enteritis [18,39,40].

Increasing attention is directed toward masked or modified forms of DON-3-glucoside and DON-sulfates. These modified forms often escape from conventional standard detection methods but are hydrolyzed in the digestive tract, releasing bioactive DON and contributing to hidden toxicity [15,27]. Their presence complicates accurate risk assessment and demonstrates the limitations of traditional detection techniques.

### 3.3. Fumonisins

FBs, particularly fumonisin B1 (FB_1_) produced by *Fusarium verticillioides* and *Fusarium proliferatum*, are commonly present in maize and its by-products. FB_1_ represents a significant mycotoxin concern in poultry production due to its immunotoxic and hepatotoxic properties [30]. FB_1_ inhibits ceramide synthase and alters sphingolipid metabolism, which is essential for maintaining cell membrane integrity and normal cell signal transduction and liver functions. This biochemical disruption leads to oxidative stress, cytotoxicity and apoptosis [15,41,42]. According to regulatory guidelines, the FDA recommends limits of a maximum of 50 mg/kg FBs concentrations in finished poultry feed and 15 mg/kg for breeding chickens, while the EU establishes a general limit of 20 mg/kg [7]. However, global surveillance studies indicate that FBs concentrations frequently exceed these thresholds in regions with high reliance on maize and inadequate post-harvest storage and handling practices. Exposure to FBs at levels of 75–400 mg/kg over a one-month period has been linked to reduced growth, decreased feed intake, and organ damage [34,38]. Clinically, birds exposed to FBs exhibit an 8–10% reduction in growth performance, decreased egg production, diarrhea, and lameness. These signs correspond with histopathological lesions characterized by hepatic and renal degeneration, vacuolation, and necrosis [28,34].

### 3.4. Zearalenone

ZEA, produced mainly by *Fusarium graminearum*, is an estrogenic mycotoxin that binds to estrogen receptors because its chemical structure closely resembles that of 17β-estradiol. This receptor interaction leads to hyperestrogenism and a wide range of reproductive disturbances in chickens, including follicular degeneration, testicular atrophy, infertility and irregular laying cycles, along with a 10 to 15% reduction in egg production and hatchability. ZEA exposure also increases embryo mortality and decreases growth performance in chicks [28,43,44].

### 3.5. Ochratoxin A

Ochratoxins, primarily Ochratoxin A (OTA), are produced by *Aspergillus ochraceus*, *Aspergillus niger*, and *Penicillium verrucosum*. OTA is a potent nephrotoxin and also exhibits immunosuppressive, teratogenic and mutagenic properties [45]. Because of its significant impact on poultry health, the EU recommends a maximum OTA concentration of 0.1 mg/kg in poultry diets [7].

Exposure to OTA at 2 mg/kg in feed results in decreased weight gain and egg production, along with increased water intake, diarrhea, anemia, poor feathering and increased mortality [46]. Young birds are highly sensitive because their immune organs, including the thymus and bursa, are not yet fully developed [47]. Necropsy commonly reveals enlarged kidneys and liver. Histopathological evaluation of affected organs shows pronounced tubular degeneration, glomerular damage, fatty infiltration, vacuolar degeneration, and lymphoid depletion in the thymus and bursa [48]. These lesions reflect the combined nephrotoxic and immunotoxic actions of OTA. In broilers, dietary exposure to 3.2 or 6.4 mg/kg OTA produces significant reductions in antioxidant enzyme activity, further exacerbating oxidative stress and cellular damage [49].

### 3.6. T-2 and HT-2 Toxins

T-2 and HT-2 toxins, classified as type A trichothecenes produced by *Fusarium* species, are among the most potent mycotoxins affecting poultry. These toxins cause severe damage to the mucosal surfaces of the oral cavity and gastrointestinal tract (GIT). T-2 toxin induces oxidative damage of cell membranes and DNA, resulting in increased apoptosis in broiler chickens [32]. The recommended T-2 and HT-2 toxin levels in poultry feed are 0.25 mg/kg according to EU guidelines [38].

Exposure to these toxins produces characteristic lesions in the oral cavity and digestive system, including ulceration, necrosis, and epithelial sloughing. Immune suppression is also prominent, as T-2 and HT-2 toxins impair both humoral and cellular immune responses. Clinically, affected birds exhibit reduced feed intake and body-weight gain, reflecting the combined impacts of gastrointestinal damage, metabolic disruption, and immune suppression [13,15,17].

The regulatory limits and maximum permissible concentrations established by the United States Food and Drug Administration (FDA) and the European Union (EU) for the major mycotoxins discussed in this review are summarized in Table 1.

### 3.7. Central Mechanisms Involved in Mycotoxin-Induced Cellular and Immune Dysfunction

Multiple mycotoxins frequently coexist in poultry feed at low or subclinical levels, and their combined exposure often results in additive or synergistic toxic effects. These interactions contribute to reduced growth performance, increased feed conversion ratio (FCR), immunosuppression, disruption of gut integrity, and heightened susceptibility to infectious diseases [7].

### 3.8. Oxidative Damage and Mitochondrial Dysfunction

A central mechanism shared among major mycotoxins, including AFB_1_, T-2, DON, FB_1_, and OTA, is the induction of oxidative stress. These toxins increase the production of reactive oxygen species (ROS) and impair the antioxidant defense system, such as the nuclear factor erythroid 2-related factor 2 (Nrf2)/Kelch-like ECH-associated protein 1 (Keap1) signaling pathway [30,32,53]. Excess ROS disrupt mitochondrial function, deplete antioxidant enzymes including superoxide dismutase, catalase, and glutathione peroxidase, and trigger damage to proteins, lipids, and nucleic acids [49]. Mitochondrial injury and increased ROS promote apoptosis through elevated Bax/Bcl-2 ratios and activation of caspase-3, caspase-9, and poly(ADP-ribose) polymerase 1 (PARP-1) [54,55].

### 3.9. Gut Integrity Disruption and Microbial Dysbiosis

Mycotoxins also share common effects on intestinal structure and barrier integrity. Reduced expression of tight junction proteins such as occludin or zona occludin increases paracellular permeability, facilitating translocation of pathogens into systemic circulation [56]. Damage to microvilli and structural proteins like ACT-5 further impairs nutrient absorption [57]. These alterations collectively promote dysbiosis, decreasing beneficial bacterial populations such as *Lactobacillus* and *Bifidobacterium* while favoring opportunistic pathogens including *Escherichia coli* and *Clostridium perfringens* [32]. Chronic exposure to subclinical concentrations of multiple mycotoxins can also significantly modify microbial diversity, short-chain fatty acid production, and metabolic pathways [32].

### 3.10. Impairment of Innate and Adaptive Immunity

Immune suppression is another hallmark of multiple mycotoxin exposure. AFB_1_, OTA, and T-2 toxins commonly decrease the number and functionality of innate immune cells, including neutrophils, macrophages, dendritic cells, and natural killer cells [58]. They impair lymphocyte proliferation and suppress interleukin-2 (IL-2) and interferon-γ (IFN-γ), and decrease the efficacy of vaccination [59]. OTA and T-2 contamination have been associated with reduced antibody titers, including a 10.4% reduction against Newcastle disease virus (NDV) [32]. Overall, these mechanisms compromise both innate and adaptive immunity, increasing susceptibility to secondary infections [60].

## 4. Masked Mycotoxins: Emerging Concerns in Feed Safety and Poultry Health

Modified or masked mycotoxins form through several biological, chemical, and feed and processing-related mechanisms (Table 2).

**Figure 2:**
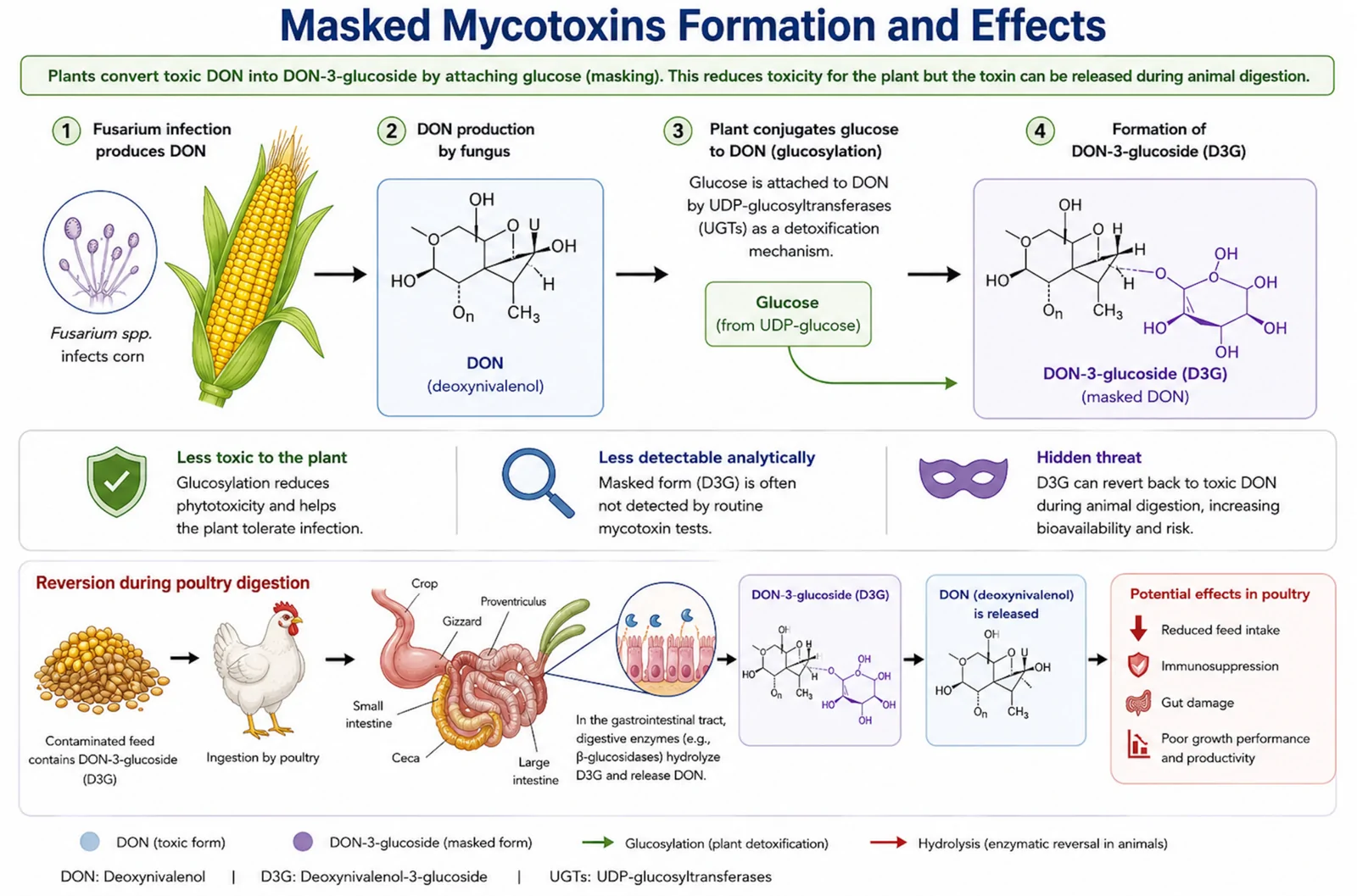
Formation of masked mycotoxins and their activation once they enter animal systems. Created in BioRender. Publication License: Shanmugasundaram, R. (2026) https://BioRender.com/bvckt43 accessed on 15 June 2026.

Plants may transform mycotoxins as part of their defense response against invading toxigenic fungi, while fungi themselves can alter toxin structures during host colonization [61]. These modified mycotoxins are often conjugated with carbohydrates, sulfates, or proteins, which alter their physicochemical properties like molecular structure, solubility, and polarity [62]. As a result, masked mycotoxins often escape detection by routine analytical techniques, including HPLC and LC-MS/MS. Once inside the animal digestive system, however, they can be hydrolyzed and release their biologically active parent compounds, leading to underestimation of true exposure levels and contributing to hidden toxicity [27] (Figure 2).

## 5. Economic and Global Dynamics of Mycotoxin Exposure in Poultry

### 5.1. Economic Implications for Poultry Production

The biological effects caused by mycotoxins translate directly into significant financial losses for poultry producers [63]. Mycotoxin exposure contributes to a 5–15% increase in FCR, reduced growth rates and egg production, increased morbidity, higher carcass condemnation due to lesions, and higher mortality, all of which increase production costs [15]. Trade rejections associated with mycotoxin residues further exacerbate the conditions, with estimated global annual losses ranging from USD 1.3 to 2.5 billion. Additional expenses arise from the use of feed additives such as binders, specialized detoxification supplements, and disposal of contaminated feed materials [28]. Although acute toxicosis is well recognized, the hidden cost of chronic or subclinical mycotoxin exposure is often overlooked in routine farm operations. Such low-level contamination can quietly impair performance over time, sometimes exceeding the economic impact of acute outbreaks. Therefore, it is necessary to take effective mitigation strategies to reduce financial and production losses [34].

### 5.2. Evolving Global Trends Shaping Mycotoxin Risk

Mycotoxin contamination patterns continue to shift over time globally, influenced by changing climatic and environmental conditions [64]. Climate change plays a major role in this shift, altering rainfall patterns, temperature profiles, and humidity levels that support fungal growth and toxin production [26]. Warm and humid tropical regions are experiencing increased AFL challenges, whereas cooler temperate zones are encountering increased incidences of FBs and DON contamination [65]. Unpredictable rainfall, prolonged drought, and heat stress further favor fungal growth both during cultivation and post-harvest storage [66,67]. Due to huge demand for raw feed ingredients, sourcing raw materials from diverse geographical regions has been led, particularly during periods of scarcity. This practice increases the likelihood of multiple species of fungal contamination and introduces region-specific mycotoxin profiles into the feed supply chain [15]. Recent global mycotoxin surveys indicate frequent co-occurrence of DON, FBs and ZEA in finished poultry feeds [68]. Such combinations increase the risk of additive or synergistic toxic effects, intensifying the overall threat posed to poultry health and production [15,63].

## 6. Mycotoxin Control Strategies in Poultry Production

Effective mycotoxin management in poultry production relies on a comprehensive strategy that integrates preventive, managemental, and biological interventions. Because mycotoxins may develop at multiple points along the feed chain, including cultivation, harvesting and storage, they pose persistent challenges to both food safety and economic sustainability. These consequences emphasized the importance of implementing efficient intervention strategies to mitigate the adverse effects associated with mycotoxin exposure.

### 6.1. Pre-Harvest Control Measures

Pre-harvest mycotoxin mitigation focuses on good agricultural practices designed to limit fungal infection and toxin production. Strategies include cultivating pest- and fungi-resistant varieties, implementing crop rotation and regular tillage, maintaining adequate irrigation to reduce drought stress, and applying effective insect and disease control measures [69,70,71]. However, these pre-harvest measures may be limited by high implementation costs, unpredictable climate conditions, and reduced yields associated with resistant varieties.

### 6.2. Harvest Control Measures

Factors during harvesting such as irregular rainfall, delays in harvest, mechanical damage to kernels, and high grain moisture significantly influence fungal growth and subsequent toxin accumulation. Recommended practices include timely harvest, appropriate machinery speed, and minimizing kernel damage. However, these practices can be difficult to implement consistently in large-scale grain production systems. Consequently, post-harvest management becomes critical for preventing fungal growth and mycotoxin formation [72].

### 6.3. Post-Harvest Mycotoxin Detoxification Methods

Post-harvest interventions are flexible and play a major role in maintaining poultry feed safety. They include physical, chemical and biological methods aimed at reducing or inactivating mycotoxins [28,73].

#### 6.3.1. Physical Methods of Detoxification

##### Sorting and Cleaning

Sorting and cleaning remove visibly damaged kernels and foreign materials, which often contain higher mycotoxin concentrations. These approaches can significantly reduce toxin levels without introducing chemical residues but are limited by the fact that mycotoxins are often invisible and unevenly distributed [74,75].

##### Drying and Controlled Storage

Drying decreases the susceptibility of grains to fungal infections or pests, as it decreases moisture levels and water activity. Limitations include high fuel costs, inconsistent moisture removal, and inability to eliminate existing mycotoxins. Cool, dry, well-ventilated storage conditions slow fungal growth but require substantial infrastructure and management [76,77].

##### Heat Inactivation

Processing techniques such as extrusion, pelleting, roasting, and boiling can reduce heat-labile mycotoxins. However, AFB_1_, OTA, ZEA, and DON are largely heat-stable, limiting their effectiveness [32]. Detoxification depends on food matrices, temperature, and duration, and toxin type [78]; excessive heat may reduce nutrient quality and sensory characteristics of food products [74,75].

##### Irradiation

Ionizing radiation (X-rays and gamma (γ) rays) and non-ionizing radiation (ultra-violet (UV), microwave, infrared, and radio waves) can inhibit fungal growth or structurally degrade mycotoxins [74,78]. However, higher irradiation doses are often required for the complete degradation of toxins, which may alter nutritional quality, affect microbial ecology, or reduce overall consumer acceptability, limiting its applicability in large-scale [32,74,78,79].

##### Cold Plasma Treatment

Cold plasma is the fourth state of matter, disrupts fungal cell walls, inhibits spore formation, and reduces toxin production. It leaves minimal residues and typically does not impair food quality. Despite promise, further research is needed to optimize parameters for large-scale application [74,80,81,82,83].

##### Adsorption

Usage of feed-grade toxin binders remains one of the most common strategies for reducing gastrointestinal absorption of mycotoxins [32,84]. Clay-based binders are more effective against polar toxins such as AFLs, but show reduced efficacy against less polar compounds such as DON and ZEA [85]. Binder performance depends on surface area, charge distribution, and affinity for specific toxin functional groups. As a result, efficacy varies considerably among mycotoxins, making it essential to evaluate binders targeted to specific toxin profiles [74,86].

#### 6.3.2. Chemical Methods of Detoxification

Chemical detoxification can significantly reduce mycotoxin levels in raw feed materials, but these approaches may leave chemical residues, alter sensory attributes, and have regulatory limitations. Although effective, they must be evaluated carefully for safety and practicality [86].

##### Alkaline Treatment

Under high temperature and pressure, basic compounds such as ammonia, calcium or sodium hydroxide open lactone rings of AFL by hydrolysis and destroy epoxide rings of DON. However, the chance of forming masked forms and the risk assessment of chemical residues after treatment limit their use [74,87].

##### Ozonation

Oxidizing agents such as ozone, hydrogen peroxide, calcium or sodium hypochlorite can degrade AFLs, DON, and ZEA. Nevertheless, treatment efficiency depends on ozone concentration and exposure time, while excessive ozonation causes protein denaturation, lipid oxidation and unwanted changes in feed quality [87,88].

##### Organic Acid

Organic acids such as citric and propionic acids chemically modify mycotoxins into less toxic derivatives. Although effective, high acid concentrations can alter feed pH, nutritional value, and sensory characteristics of feed products [74,89].

#### 6.3.3. Biological Detoxification Methods

Biological detoxification has gained attention because physical and chemical approaches act externally and cannot reverse physiological damage caused by mycotoxins. Microorganisms and their enzymes provide an environmentally friendly detoxification option and can degrade toxins through adsorption or biotransformation (Table 3) [86].

##### Probiotics

Beneficial bacteria such as *Lactobacillus*, *Bacillus*, *Enterococcus*, and yeasts like *Saccharomyces cerevisiae* detoxify mycotoxins by binding to cell-wall components or transforming into less-toxic metabolites. These microorganisms are generally considered safe and environmentally friendly. Their efficacy, however, varies among strains, toxin types, and environmental conditions [28,74].

##### Enzymatic Detoxification

Although microorganisms can degrade mycotoxins effectively, many strains are unable to survive or remain active in the gastrointestinal environment. Their enzymes, such as oxidases, peroxidases, and reductases, can function independently and break down AFB_1_, ZEA, FBs and DON. Enzymatic detoxification is environmentally safe and highly specific, but its practical application is limited by enzyme stability, sensitivity to feed-processing conditions and cost of commercial production [74,75].

Despite progress in physical, chemical, and biological detoxification methods, no single approach is broadly effective or economically feasible for complete mycotoxin removal from poultry feed [30]. This limitation emphasizes the need for predictive modeling and advanced risk management strategies that can identify contamination risks early and support proactive intervention rather than relying solely on post-contamination detoxification.

## 7. Challenges with Current Analytical Quantification of Mycotoxins

The heterogeneous distribution of mycotoxins within grains and feed samples is a major challenge to reliable detection. Localized “hotspots” typically associated with areas of high moisture have substantial fungal growth and high mycotoxin concentrations [105]. Collecting a truly representative sample is a major challenge if sampling is limited or improperly stored. These variations lead to non-representative samples and inaccurate quantification [106]. Therefore, to ensure reliable detection, representative sampling strategies, including sample mixing, multiple subsamples, and sample pooling, are essential to ensure meaningful assessment of contamination levels [61].

Conventional analytical techniques for mycotoxin detection include ELISA, radioimmunoassay, chromatographic methods and mass spectrometry. Immunological assays, particularly lateral flow devices and ELISA, are widely used due to their relative simplicity, speed, and cost-effectiveness for routine screening [107]. However, immunological detection methods have limitations due to antibody cross-reactivity with modified metabolites and low sensitivity toward masked or modified toxins and matrix interference effects that reduce sensitivity and specificity [61]. As a result, immunoassay findings often require confirmation using chromatographic methods [22].

Chromatographic techniques such as HPLC and LC-MS/MS remain the gold standard for accurate quantification and identification of known mycotoxins [23,24,25]. HPLC commonly utilizes UV or fluorescence detectors [108], whereas LC-MS/MS offers more selectivity and sensitivity by detecting analytes based on mass-to-charge ratios [22]. Despite their reliability, these technologies require highly trained personnel, specialized laboratory facilities, complex operational procedures and high analytical costs [12,109]. Even when optimized, chromatographic methods may fail to detect low-level co-contamination. For example, UHPLC-MS/MS-based limits of detection (LOD) and limits of quantification (LOQ) values vary widely across mycotoxins. AFB_1_ and ZEA typically exhibit LODs of 0.25 ng/g, with LOQs of 0.5 ng/g and 1.0 ng/g, respectively. OTA, T-2, and HT-2 toxins showed LODs of 2.0 ng/g, while the corresponding LOQs were 4.0 ng/g for OTA and T-2, and 8.0 ng/g for HT-2. While FB_1_ presents much higher LODs of 15.0 ng/g and an LOQ of 30.0 ng/g, whereas DON showed an LOD of 20.0 ng/g and an LOQ of 100.0 ng/g [110]. Mycotoxins present below these thresholds may escape chromatographic detection yet still exert biological effects in birds, creating hidden risks that complicate risk assessment.

The presence of masked/modified mycotoxins creates additional analytical challenges. Structural modifications alter polarity, solubility, and antigenic sites, weakening antibody recognition [27]. ELISA-based methods may therefore under- or overestimate contamination due to inconsistent cross-reactivity, while chromatographic methods lack validated reference standards and often show low sensitivity for modified mycotoxins. Furthermore, conventional analytical methods are optimized for known mycotoxins [111], and their limits of detection (LOD) are also relatively low when applied to complex feed matrices [63,112]. Modified forms have altered structure and polarity compared to the parent compound, leading to poor extraction from feed with regular solvents and a lack of analytical standards for accurate quantification [113]. These limitations demonstrate the need for emerging technologies, such as biosensor-based sensing systems and predictive modeling tools, to improve real-time detection and early risk forecasting of mycotoxin outbreaks [114]. To emphasize the differences between conventional analytical approaches and model-assisted prediction tools, a comparative summary of their major strengths and limitations is provided in Table 4.

## 8. Machine Learning

Machine learning has emerged as a powerful data-driven technology capable of addressing several challenges associated with mycotoxin detection. ML can analyze high-dimensional datasets and identify subtle patterns that conventional analytical methods often fail to detect mycotoxin contamination [12]. Integrating ML into mycotoxin research, scientists can improve detection accuracy, enhance pattern recognition, and provide early warnings of potential mycotoxin risks. Modern analytical methods increasingly adopt ML and deep learning (DL) algorithms to enhance precision, speed, and affordability in mycotoxin detection. These computational tools support improved decision-making throughout the food and feed supply chain [12,127].

ML refers to an advanced scientific tool in which algorithms learn from complex datasets to recognize patterns and make decisions autonomously, without being rigorously coded for every task [128,129,130]. ML models are generally classified into three types based on how they learn: supervised, unsupervised, and reinforcement learning mechanisms. In supervised learning (SL), models are trained using a labeled dataset in which each input is paired with a known output. By learning these relationships, the model can later predict missing or unknown outputs for new data that were not part of the training set [131]. In unsupervised learning (UL), algorithms process unlabeled datasets and independently identify underlying structures, clustering patterns, or relationships within the datasets. After training, the model can categorize or organize new inputs based on learned patterns [129]. In reinforcement learning (RL), the model learns through repetitive interactions with its environment that lead to a desired goal. Algorithms receive rewards or penalties for their actions and gradually develop strategies that maximize cumulative reward [12,128].

ML models used in mycotoxin research incorporate various data types, including multispectral or hyperspectral images of feed ingredients [132,133], environmental data, and sensor-based outputs such as electronic nose (E-nose) signals. These tools enable improved detection, classification, and forecasting of mycotoxin contamination in grains and finished feeds [134]. By learning relationships between environmental conditions, fungal activity, chemical nature, and feed characteristics, ML systems offer promising opportunities for early risk prediction and real-time monitoring within poultry production systems.

### 8.1. Spectral and Image-Based Data

**Figure 3:**
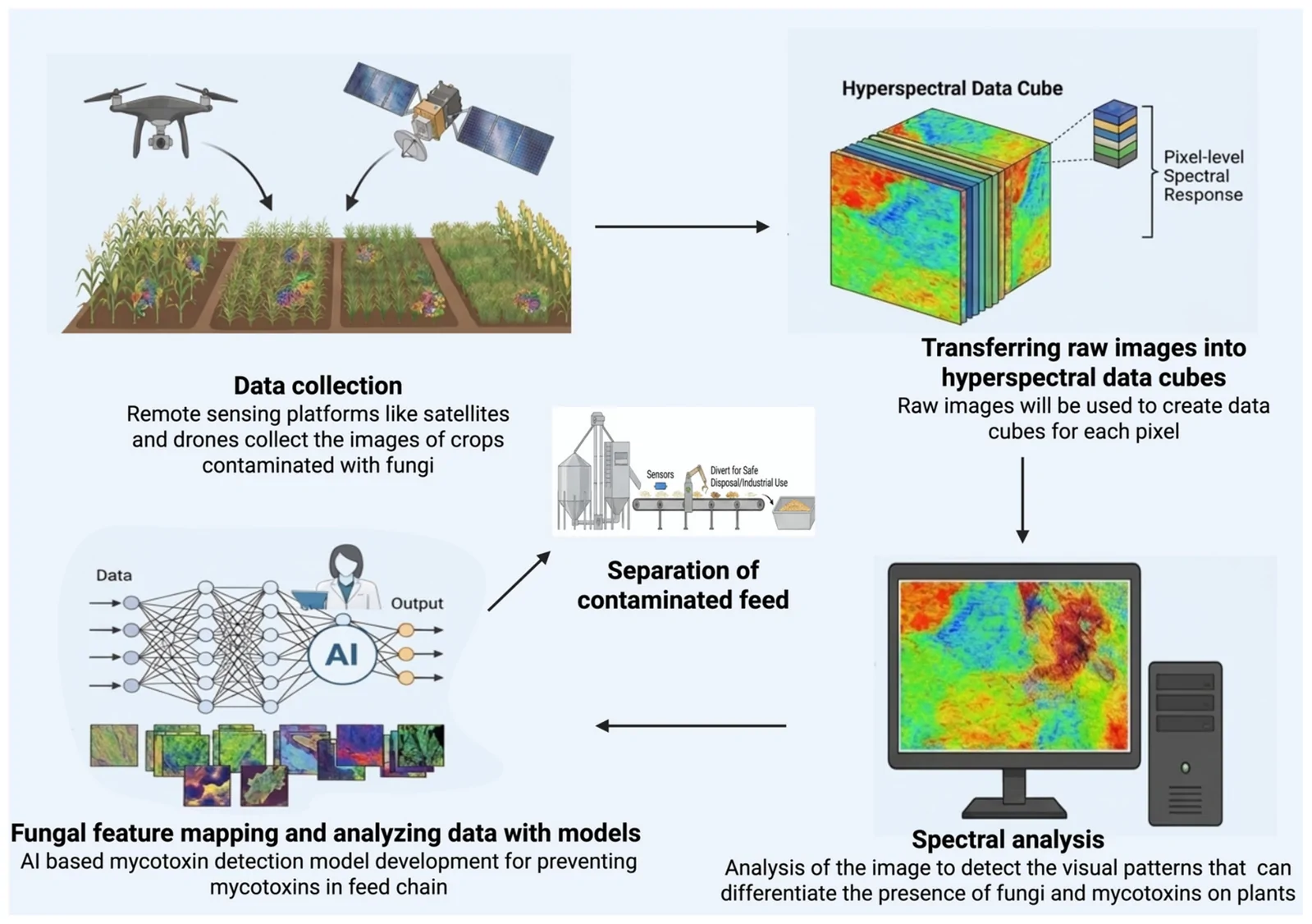
AI-assisted workflow for early mycotoxin detection using remote sensing and hyperspectral imaging technologies. Created in BioRender. Shanmugasundaram, R. (2026) https://BioRender.com/hy6jgpt accessed on 15 June 2026.

Spectral imaging represents a non-invasive analytical approach that includes infrared spectroscopy, hyperspectral and multispectral imaging [135]. Fluorescence hyperspectral sensors have shown promise for rapid and cost-effective detection of AFL contamination in corn kernels [136]. ML models can utilize spectral data to identify chemical changes associated with fungal growth or toxin production (Figure 3), enabling early prediction of mycotoxin risk [137].

Hyperspectral imaging has been used to identify moldy grains and estimate DON concentration in poultry feed and litter by analyzing carbon and nitrogen concentration using hyperspectral imaging combined with partial least squares regression models [138]. However, one of the major limitations of spectral-based ML approaches is the scarcity of naturally contaminated samples across diverse toxin concentrations. This restricts model training and validation, as artificial contamination does not fully reproduce the complexity of natural fungal infection [133]. Additionally, spectral properties of artificially spiked samples often differ from naturally contaminated grains, causing inconsistencies in model performance, which indicates the challenge of recreating the full range of real-world toxin levels [139].

### 8.2. Sensors and Internet of Things (IoT)-Based Data

The integration of sensor technologies with Internet of Things (IoT) platforms represents a major advancement in mycotoxin monitoring and prevention. During grain production, storage, and transportation, networked sensors can continuously track environmental conditions and automatically transmit real-time alerts when values exceed safe thresholds [140,141]. This continuous surveillance enables early identification of conditions that favor fungal growth and mycotoxin formation. In storage facilities such as silos, smart sensors can monitor microclimatic parameters, including temperature, humidity, CO_2_ levels, and water activity, providing a preventive approach for detecting shifts that increase contamination risk [130,142]. When undesirable changes are detected, corrective measures such as adjusting aeration, improving drying, or isolating high-risk batches can be implemented promptly to reduce toxin development [141]. However, these climate-driven shifts also affect the adaptability of machine-learning prediction models, which often require recalibration when applied across different climatic regions due to changes in fungal ecology, storage conditions, and contamination patterns. Because most ML models are trained on region-specific datasets, their predictive accuracy may decline when used in different climate zones unless they are retrained or adjusted to account for environmental variability. IoT-enabled monitoring systems strengthen feed-safety management by offering continuous data streams, automated alerts, and rapid decision support. These features make sensor-based technologies particularly valuable for early detection and prevention of mycotoxin hazards in poultry production environments.

### 8.3. Machine Learning Approaches for Mycotoxin Detection

ML models such as neural networks, random forests, gradient boosting, and support vector machines are increasingly applied to mycotoxin detection and management [133,138,143]. A brief overview of key algorithms, including their strengths and limitations, is provided below.

#### 8.3.1. Artificial Neural Networks

Artificial Neural Networks (ANN) are ML algorithms inspired by the structure and function of the human brain. They process information through multiple layers of interconnected neurons, enabling the model to learn complex, non-linear relationships, recognize patterns, and generate predictions from multidimensional datasets [144]. Input variables enter through the input layer, pass through one or more hidden layers where weighted computations and non-linear transformations occur (commonly via activation functions such as ReLU), and yield final predictions at the output layer [12,130,145]. During training, prediction error is minimized by iteratively adjusting neuron weights. Overfitting is commonly controlled using regularization approaches, such as through L2 regularization [146]. The output of an individual neuron can be expressed mathematically as the weighted sum of its inputs passed through a non-linear activation function:

$$y=\;f\left(\sum_{i=1}^{n}w_{i}x_{i}\;+\;b\right)$$

where x_i_ represents the input variables, w_i_ the corresponding weights, b the bias term, *n* the number of inputs, and f the non-linear activation function (e.g., ReLU or sigmoid).

The non-linear and multifactorial nature of mycotoxin contamination is efficiently handled by ANNs, particularly suitable for predictive modeling. Reliable prediction of toxin contamination by ANN with integration of various input variables like climate factors, grain composition, and storage environments [12]. ANNs have been used to forecast AFL formation under varying temperature and humidity conditions, estimate DON accumulation in cereal-based feeds, and predict toxin transfer during feed manufacturing. They have also been applied to identify AFL and FB contamination in corn at harvest. Even though many ANN-based studies report moderate performance, for example, 75% accuracy in some mycotoxin detection tasks, indicating the need for further research to clarify complex toxin interactions [128].

Convolutional neural networks (CNNs) are a specialized type of ANN designed for image-based processing and pattern recognition [130]. Unlike traditional ANNs, CNNs incorporate convolutional, activation, pooling and fully connected layers that automatically extract spatial and hierarchical features from multidimensional image data, such as edges, textures, and shapes [131,147]. Because CNNs use local feature learning and weight sharing, they achieve high accuracy and computational efficiency. CNNs are widely used in hyperspectral imaging for early mycotoxin detection [148]. CNNs are used for fungal infections or chemical markers associated with toxin production, often before mold becomes visible [149].

Neural network (NN) models face several limitations in mycotoxin research. When datasets are small or imbalanced, NNs require careful calibration to prevent overfitting and must undergo rigorous training and validation to ensure reliable performance [12,150]. The limited availability of labeled datasets further restricts model accuracy and increases the risk of overfitting. In addition, training large NNs requires significant computational resources, and the complexity increases overall processing cost [151]. To address these constraints, NNs are often combined with more interpretable algorithms, such as random forest or used with ensemble frameworks to improve robustness and predictive accuracy under limited data conditions [152]. CNN-based systems can effectively identify fungal contamination, but they may not distinguish toxin-producing fungi from non-toxigenic ones, reducing specificity in practical applications [153].

#### 8.3.2. Integration of Neural Networks with Electronic Nose Systems

An electronic nose (E-nose) system consists of sensor arrays designed to detect volatile chemical compounds present in gases. When combined with NN algorithms, E-nose platforms have been successfully applied to rapid screening of AFLs, DON and FBs in corn, achieving detection accuracies of approximately 77% [12]. Recent work also demonstrates that coupling an E-nose with NNs and ensemble learning algorithms can accurately identify ZEA-positive pet food samples [134]. Integrating biosensors with ML models improves on-site mycotoxin detection capability. Biosensors capture chemical, electrical or optical signals associated with fungal growth or toxin contamination, and ML algorithms immediately analyze these sensor outputs to generate rapid classification or risk alerts. This combined approach enables fast, portable, and affordable screening solutions ideal for feed mills, storage sites and field environments.

While E-nose technologies are highly effective for distinguishing contaminated from non-contaminated materials, they still have limitations. Sensitivity is generally lower than that of conventional laboratory-based methods such as ELISA or LC-MS/MS, and current E-nose systems are not yet suitable for precise quantification of mycotoxin concentrations [154]. As a result, E-nose platforms are best suited as preliminary screening tools within broader mycotoxin surveillance programs.

#### 8.3.3. Random Forests

Random forest (RF) is one of the most widely used machine learning algorithms for classification and regression tasks in mycotoxin research [155,156]. RF models are composed of an ensemble of decision trees, where each tree is built from a randomly selected subset of the dataset. A typical decision tree consists of a root node, several branching decision nodes, and terminal leaf nodes that represent final outputs [12,130]. The combined predictions from all trees generate a more accurate and stable ensemble output [127]. RF algorithms have been applied to classify feed ingredients, including separating contaminated and uncontaminated corn samples using infrared spectroscopy, which supports feed-safety monitoring and mitigation strategies [157]. RF models are also commonly used for predicting mycotoxin occurrence because of their reliability and ability to detect important risk contributors [127]. By analyzing multiple environmental and production-related features such as weather conditions, crop growth stage, grain moisture content, and storage duration, RF models can identify complex patterns linked to toxin formation [12]. For regression tasks, the ensemble prediction is obtained by averaging the outputs of all individual decision trees in the forest:

$$\hat{y}=\frac{1}{T}\sum_{t=1}^{T}h_{t}\left(x\right)$$

where ŷ is the ensemble output, T the total number of trees, and h_t_(x) the prediction generated by the tth tree for input x. For classification tasks, the ensemble prediction is determined by majority voting among the trees.

The RF models are robust and resistant to overfitting, and capable of handling heterogeneous datasets, making them well suited for mycotoxin prediction models. However, their performance may require substantial computational resources when dealing with very large datasets or numerous input variables. Interpretability can also be limited in complex RF models. To address this, feature-importance metrics such as mean decrease in accuracy (MDA) and Gini importance are often used to identify the most influential variables contributing to model predictions [127,158].

#### 8.3.4. Gradient Boosting

Gradient boosting (GB) is an ensemble learning technique in which multiple weak learners are trained sequentially, with each model attempting to correct the errors of the previous one. In this approach, the output of the first decision tree is used to guide the training of subsequent decision trees, and the final prediction is generated by combining the outputs of all learners [127]. Key hyperparameters, including the number of weak learners, the learning rate, and the maximum depth of each tree, strongly influence model accuracy, complexity, and the risk of overfitting [159]. Increasing the number of learners often improves predictive performance but also raises computational demands, whereas a lower learning rate generally improves model generalization [12].

The additive, sequential nature of gradient boosting can be described mathematically as

$$F_{m}\left(x\right)=F_{m-1}\left(x\right)+\nu \cdot h_{m}\left(x\right)$$

where F_m_(x) represents the model prediction after the mth boosting iteration, F_m−1_(x) the prediction from the previous iteration, h_m_(x) the newly added weak learner based on residual error, and *ν* the learning rate that determines how much each successive learner contributes to the final prediction.

GB is effective for processing complex high-dimensional datasets and is highly effective for mycotoxin risk prediction and classification tasks. However, the sequential nature of GB results in significant computational resources and noisy datasets may increase the likelihood of overfitting if hyperparameters are not carefully optimized [12].

#### 8.3.5. Support Vector Machines

Support vector machines (SVMs) are widely used machine learning algorithms for regression, classification and outlier detection [160]. An SVM constructs an optimal decision boundary, known as a hyperplane, within a high-dimensional feature space to maximize the separation between distinct classes of data [127,161]. Kernel functions enable SVMs to transform input data—such as near-infrared spectra, environmental parameters, or sensor outputs—into higher-dimensional spaces in which contamination patterns become more easily distinguishable [162]. The SVM decision function for classifying a new observation is given by

$$f\left(x\right)=sign\left(\sum_{i=1}^{n}\alpha_{i}y_{i}K\left(x_{i},x\right)+b\right)$$

where α_i_ are the learned weight coefficients, y_i_ the class labels of the training samples, K (x_i_, x) the kernel function that projects the data into a higher-dimensional feature space, b the bias term, and sign (·) the function that assigns the predicted class from the sign of the output.

Effective class separation depends strongly on kernel selection, as each kernel function determines how input data are projected into higher-dimensional space [163]. When datasets exhibit high noise levels or considerable overlap between contaminated and uncontaminated groups, SVM performance can decline because the model struggles to establish a clear boundary [164]. As a result, misclassification becomes more likely in ambiguous or “gray zone” regions where classes are not well separated. Compared with SVMs, ensemble models such as random forests or deep learning approaches may perform better on large-scale, highly non-linear datasets, owing to their flexibility and capacity to learn complex patterns.

### 8.4. Machine Learning Applications in Poultry Mycotoxin Surveillance

Early risk assessment of fungal growth and mycotoxin production in poultry feeds has increasingly benefited from ML approaches such as SVM, RF, logistic regression (LR) and GB [109,143,165]. Models trained on environmental and feed-storage variables have successfully predicted AFB_1_, DON, and OTA contamination in fresh eggs. Among various regression and classification algorithms, ensemble-based models, particularly random forest and extra trees, demonstrated superior predictive performance, achieving classification accuracies exceeding 97%. These results highlight the potential of machine learning as a rapid and cost-effective tool for mycotoxin risk assessment in poultry products [143]. Behavior-based ML applications have also shown promise for early detection of mycotoxin toxicity. An AI-assisted monitoring system developed by [165] used cameras and wearable tri-axial accelerometers to evaluate behavioral changes in broilers exposed to AFB_1_. Affected birds exhibited more sitting behavior and reduced feeding, drinking, and walking activities. Among the ML algorithms tested, the Gradient Boosting Decision Tree (GBDT) achieved the highest classification accuracy (~97%), followed by Random Forest (~96%). Similar studies demonstrate that AI-driven behavioral analysis provides a non-invasive and sensitive method for detecting early signs of aflatoxicosis in poultry production systems [166].

Multi-mycotoxin-contaminated maize grains were screened through fluorescence spectroscopy and machine learning models such as LR and DT. The model predicted AFB_1_, DON, ZEA and FBs with 73%, 91%, 86% and 95%, respectively. Results of the model were cross-validated with a binary and multi-label classifier [167]. Masked mycotoxins can be predicted by the MycotoxinDB database model, which works on 78,000 reaction rules to predict possible chemical and biological transformations of parent toxins. This model achieved 87.51% recall and predicted 48 masked forms of DON, seven of which were experimentally confirmed in wheat using LC–MS/MS [123].

### 8.5. Machine Learning as a Predictive Shield: Algorithms Transforming Mycotoxin Forecasting

Predictive modeling offers poultry producers and feed millers the ability to anticipate mycotoxin risks before contamination occurs, enabling timely and targeted preventive interventions [168]. Conventional statistical techniques often struggle to detect relationships among multiple environmental and storage-related factors, particularly when these variables interact in complex, non-linear ways. In contrast, ML models can uncover hidden patterns, adapt to diverse datasets and generate precise predictions across different production systems [127]. With the rise in climate variability and the globalization of food supply chains, predictive ML technologies are becoming essential components of proactive approaches to food and feed safety. To emphasize variability in predictive performance across commonly used machine learning algorithms, Table 5 summarizes representative models, sample sizes, validation approaches and reported accuracy values from published studies.

### 8.6. Translational Learnings: Cross-Species AI Models and Their Relevance for Poultry

While the use of artificial intelligence-driven toxicological analysis tools is yet to be adopted by the poultry industry, their use has been demonstrated in other animal production sectors. Recent developments in sensor technology show that machine learning can be used to recognize the volatile compounds linked to ZEA contamination in pet food products [134]. Studies reported that high-risk AFB_1_-contaminated feed supplies can be detected using several ML algorithms, including extreme gradient boosting, SVM, decision trees and logistic regression [174].

Adapting such cross-species ML models for poultry requires careful calibration and validation to account for biological, physiological, and metabolic differences between species. Even so, these multispecies approaches provide valuable foundational insights, particularly in cases where poultry-specific datasets remain limited. They also support the development of sensor-based monitoring systems and intelligent detection platforms that can be tailored for poultry production environments as more relevant data become available.

### 8.7. Barriers to the Adoption of ML Models and IoT Tools

Despite substantial advancements in combined IoT technologies and ML models for mycotoxin mitigation, several barriers still limit their widespread adoption in poultry production systems. The effectiveness of ML algorithms depends heavily on the availability of high-quality, consistent datasets. Limited or inconsistent data make it difficult to train robust models and often reduce predictive performance. Variability in environmental conditions, sensor calibration challenges, and sensitivity to differences in ingredient composition can also impact the reliability of real-time monitoring systems [12]. Differences in infrastructure across farms further contribute to inconsistent implementation. Large commercial farms may have access to advanced monitoring systems, whereas smaller operations often lack the resources, connectivity, or technical support necessary to deploy IoT-based tools effectively. Regulatory frameworks also continue to require confirmation through established laboratory methods such as ELISA or LC-MS/MS, making exclusive reliance on ML-generated predictions unsuitable for official testing purposes. Additionally, high initial costs and limited technical expertise among producers can slow technology adoption. Without adequate training and support, the perceived complexity of ML and IoT systems may discourage their use. These challenges indicate the need for improved model optimization, better sensor standardization, expanded data availability, and accessible training resources to support broader implementation of ML-based mycotoxin surveillance [127,175]. Adoption on small- and medium-sized poultry farms is further constrained by high implementation costs, limited data infrastructure, inadequate automation, and shortages of technical expertise. Successful deployment therefore requires affordable, user-friendly systems, sustained technical support, and stakeholder involvement to integrate AI tools into existing farm management practices [176].

### 8.8. Practical Relevance of Machine Learning in Poultry Mycotoxin Management

In commercial poultry operations, raw materials for feed are sourced from multiple suppliers across diverse geographical regions. Feed mills routinely receive corn, soybean meal, DDGS, wheat, and various other feed ingredients produced under different climatic conditions, harvested with different techniques, and stored and transported using practices that vary widely [2]. These regional differences lead to substantial variability in the type and level of mycotoxin contamination present in individual ingredients. Although each ingredient may contain mycotoxins at levels below regulatory limits, combining several ingredients into a single feed formulation can result in a finished feed that contains multiple toxins simultaneously [7]. Poultry feed is therefore continuously exposed to mycotoxins at low levels that cannot be detected by any screening process. Machine learning is particularly useful in this context because it can detect contamination patterns that conventional analytical approaches may fail to identify. Integrating ML algorithms in feed-monitoring platforms enables continuous analysis of large volumes of real-time data. These datasets include information related to ingredient origin, storage temperature, humidity, moisture content, environmental conditions conducive to fungal proliferation, and historical contamination trends. By analyzing these variables, ML models can estimate the probability of contamination, forecast potential outbreaks, and identify high-risk batches requiring segregation before feeding [133]. Intelligent storage systems equipped with sensors provide additional support by monitoring microclimatic conditions within silos and alerting producers when parameters favor fungal growth and toxin formation [140]. AI-driven methods can also identify suspect feed ingredients requiring confirmatory laboratory testing, allowing targeted and efficient quality control. Based on ML-generated insights, producers can segregate contaminated lots, adjust storage conditions, incorporate mycotoxin deactivators, and modify feed formulations as needed. In this way, the utilization of AI and machine learning technology in poultry production systems offers a preemptive solution to the challenge of mycotoxicosis.

## 9. Conclusions and Future Perspectives

No single ML model can be considered universal or superior for all mycotoxin detection and prediction scenarios. Model performance depends strongly on the type of data available, the nature of the analytical task, environmental variability, and the complexity of contamination patterns. Although several ML algorithms have proven effective, each has its own strengths and limitations. SVMs are particularly well suited for high-dimensional datasets and situations with limited sample sizes, making them useful for spectroscopy and hyperspectral imaging analyses. RF models offer reliability, robustness to overfitting, and strong capability to identify key risk factors contributing to contamination. ANNs and deep learning architectures such as CNNs perform well when large, complex datasets are available, especially those derived from multispectral imaging, environmental monitoring, and sensor-based systems. Despite this progress, no single model consistently outperforms others across all production conditions. From a future perspective, the use of ML and spectral image-based tools in poultry mycotoxin surveillance remains promising. Future research should prioritize the development of open-source software, standardized analytical workflows, and cost-effective methods to facilitate wider adoption and reproducibility across laboratories and production environments. Integrating multiple ML models through ensemble or hybrid approaches may further improve predictive accuracy and enhance model flexibility across diverse climates, feed ingredients, and agricultural practices. Continued innovation in this field will support more proactive monitoring systems and strengthen food and feed safety in modern poultry production.

## Figures and Tables

**Table 1:** Regulatory limits and maximum permissible levels suggested by FDA and EU guidelines [50,51,52].

	The USA FDA Approved Levels			EU Regulatory Limits
	Finished Poultry Diet/Broilers	Breeders	Immature Birds	
AFL (mg/kg)	0.1	0.1	0.02	0.02
DON (mg/kg)	5	5	5	5
FBs (mg/kg)	50	15	50	20
ZEA (mg/kg)	No guidance levels established			-
OTA (mg/kg)	No guidance levels established			0.1
T2 (mg/kg)	No guidance levels established			No guidance levels established

**Table 2:** Biological and chemical mechanisms involved in the formation of modified mycotoxins.

Category	Subcategory	Modified Form
Biologically modified	Functionalized metabolites	AFB_1_-8,9-epoxide
	Conjugated by animals	AFB_1_-mercapturate
	Conjugated by plants	DON-3-glucoside
	Conjugated by fungi	3-Acetyl DON
		15-Acetyl DON
		Zearalenone-14-sulfate
Chemically modified	Thermally formed	N-carboxy-methyl-FB_1_
		14-(R)-OTA
		Iso-DON
		Nordeoxynivalenol A/B/C
	Non thermally formed	*N*-palmitoyl-HFB_1_
		DON-10-sulphonate

**Table 3:** Different mycotoxin detoxifiers used as an effective mitigation strategy against mycotoxins.

Mycotoxin Detoxifier		Target Mycotoxin	Impacts	Reference
Type	Concentration			
Lactobacillus species	0.20–0.40 g/kg	DON, AFB_1_, ZEA	Improved villus height, crypt depth, muscular thickness, and absorptive area and spleen weight, enhanced NDV antibody titers, restoration of normal gut microbiota, reduction in AFB_1_ residues in organs and down-regulated TLR-4 and upregulated Claudin-5 and MUC-2 expression	[90,91]
*Bacillus subtilis* or *Bacillus licheniformis*	6–7 log CFU/g	AFB_1_, ZEA, OTA, T-2 toxin	Increase growth performance and nutrient metabolic rates, decrease diarrhea rates and mortality for broilers, positively regulate gut microbiota, decrease lipid peroxidation, apoptosis and cytotoxicity and improve Avian Influenza, NDV and IB antibody titers	[92,93,94]
*Saccharomyces cerevisiae*	0.75–1.50 g/kg	DON, OTA, T-2 toxin	Restored normal liver histology, reducing intestinal inflammation and villus damage, and significantly lowering residual AFL levels in the liver	[90,93,95]
Aflatoxin B_1_-degrading enzyme	1 g/kg	AFB_1_	Improved growth performance and decreased AFB_1_ residues in organs	[33,96]
Bentonite	4 g/kg diet	AFB_1_, OTA	Along with 4% zeolite bentonite, it increased DWG but decreased FCR, improved dressing percentage, apparent CP digestibility, liver weight and significantly reduced AFB_1_ residues in the liver	[84,97,98,99]
Zeolite	1–4 g/kg diet	AFB_1_, OTA, T-2 toxin	Improved FCR, ADG, glutamate dehydrogenase concentration in serum; reduced OCT concentration in kidney and AFB_1_ concentration in liver	[93,100]
Aluminosilicates	0.5–5 g/kg	OTA, T-2	Improved growth performance and serum concentrations of total protein, glucose, phosphorus, Alkaline phosphatase, creatinine phosphokinase and aspartate aminotransferase reversed intestinal damage caused by T2 toxin	[97,101,102]
Montmorillonite	0.3 g/kg	AFB_1_	Reduced AFL residues and improved growth performance	[33]
Smectite-based clay binder	2 g/kg	AFB_1_	Improved growth performance, reduced severity of lesions caused by mycotoxins in various visceral organs like the liver and kidney, and elevated IBD virus antibody titers	[103]
Yeast cell wall extract	2.0 g/kg	AFB_1_, OTA, DON, T-2, ZEA, 3-ADON	Improved immune response, liver health, and gut integrity and growth performance	[84,97,104]
Beta-glucans	5 g/kg	OTA	Improves growth performance and is effective when used along with other toxin binders	[97]

**Table 4:** Differences between traditional analytical methods and model-assisted methods.

	Conventional Analytical Methods	Model-Assisted Methods
Examples	ELISA, lateral-flow immunoassays and chromatography techniques (HPLC and LC-MS/MS) [107]	ML models combined with hyperspectral images, electronic noses, biosensors, soil and environmental monitoring data [115]
Strength	High analytical sensitivity, specificity, accuracy and quantitative reliability [116] Results are consistent and reproducible because of standard operating procedures and regulatory guidelines across various laboratories [117]	Rapid analysis of complex data and potential for high-throughput or real-time screening [118]
Limitation	Often require extensive sample preparation, trained personnel, are expensive, time-consuming and laboratory-dependent [119]	Performance depends heavily on training data quality and representativeness [120]
Multiple-mycotoxin analysis	LC-MS/MS can quantify numerous toxins simultaneously [121]	Possible when the model is trained with sufficiently diverse multi-mycotoxin data [122]
Masked mycotoxin detection	Internal standards are required and difficult to detect with LC-MS/MS methods [27]	Limited because masked forms are often underrepresented in training datasets [123]
Real-time surveillance	Generally limited by sampling, transport and laboratory turnaround [115]	Can provide continuous or near-real-time risk alerts when integrated with sensors [124]
Regulatory acceptance	Validated chromatographic methods are widely accepted for confirmation and enforcement [125]	Generally used for preliminary screening or decision support rather than official confirmation [126]

**Table 5:** Overview of machine learning models and their predictive performance in poultry and pet food studies.

Model	Sample Size	Prediction Task	Validation Strategy,	Reported Accuracy, Sensitivity, Specificity	Reference
RF, DT, SVM, k-nearest neighbors (k-NN), Gradient Boosting Decision Tree (GBDT)	216,000 sets of data points	Identified birds poisoned with AFB_1_	Confusion matrices	Accuracy for k-NN, SVM, DT, RF and GBDT is 83%, 83%, 94%, 96% and 97%, respectively, for disease identification	[165]
LR, k-nearest neighbors (k-NN), SVM, RF, XG Boost, and Multi-layer perceptron (MLP)	142 pet food samples	Predicting ZEA contamination levels in pet food	Receiver operating characteristic (ROC) curves and respective AUC values	Accuracy is 86.6% in the MLP algorithm and 77.1% to 84.8% in other models	[134]
SVM, NN, ELM (Extreme learning machine)	20 min audio files of experiment	Early disease diagnosis using rales audio sounds	ROC curves	In ELM, accuracy is 97.1%, precision is 88.1%, and recall is 81.5%. In SVM, accuracy is 97.6%, precision is 86.6%, and recall is 85.2%.	[169]
SVM, ELM	110 images data	Avian pox disease recognition	Confusion matrix and Root Mean Square Error (RMSE)	Accuracy is 92.77% and 95.01% for SVM and ELM, respectively. Sensitivity is 90.8% and 93.75% for SVM and ELM, respectively. Specificity is 94.93% and 96.59% for SVM and ELM, respectively	[170]
ANN	360 data pairs	To predict feed conversion, water consumption and cloacal temperature	Mean square error (MSE), Linear regression	R^2^ is 0.86, 0.96 and 0.84 for feed conversion, water consumption and cloacal temperature, respectively.	[171]
ANN	Records from 1136 quails	Predict and optimize slaughter body weight of quails, based on their early growth performances, sex, and egg weight	Mean standard error (MSE)	The R^2^ values were 0.9404, 0.9359, and 0.9223 for the training, validation, and testing phases, respectively.	[172]
ANN, BN, DT, SVM	150 Eggs	Dielectric spectroscopy and machine learning were used to classify poultry eggs based on freshness	Root Mean Square Error (RMSE)	ANN, BNs and SVM showed 100% accuracy, whereas DT showed 87.5% accuracy R^2^ values are 0.817, 0.906 and 0.920 for ANN, DT and SVM, respectively	[173]

## Data Availability

The original contributions presented in this study are included in the article. Further inquiries can be directed to the corresponding author.
